# Prevalence of diabetes among homeless men in Nagoya, Japan: A survey study

**DOI:** 10.1111/jdi.12943

**Published:** 2018-10-24

**Authors:** Mayumi Yamamoto, Takahiro Watanabe, Ryosuke Uehara, Ryo Horita, Tadahiro Sado, Akihiro Nishio

**Affiliations:** ^1^ Health Administration Center Gifu University Gifu Japan; ^2^ United Graduate School of Drug Discovery and Medical Information Sciences Gifu University Gifu Japan; ^3^ Department of Endocrinology and Metabolism Gifu University Hospital Gifu University Gifu Japan; ^4^ Department of Psychiatry Midori Hospital Gifu Japan; ^5^ Department of Psychiatry Yoshida Hospital Nara Japan; ^6^ Department of Psychiatry Gifu University Hospital Gifu University Gifu Japan; ^7^ Faculty of Health Promotional Sciences Tokoha University Hamamatsu Japan

**Keywords:** Diabetes mellitus, Homeless persons, Social support

## Abstract

**Aims/Introduction:**

The diabetes status of homeless people has not been elucidated because of the limited access to this population. We carried out a survey of the prevalence of diabetes and prediabetes among homeless men in Nagoya, Japan, and assessed the associations between diabetes prevalence and sociodemographic characteristics.

**Materials and Methods:**

Interviews relating to individuals’ sociodemographic background, mental illness and cognitive ability, and blood sampling were carried out for 106 homeless men (mean age 54.2 ± 12.7 years). Diabetes, prediabetes and normoglycemia were diagnosed according to the individual's hemoglobin A1c level: ≥6.5%, 6.4–6.0% and ≤5.9%, respectively. Mental illness and cognitive disability were diagnosed using the Mini‐International Neuropsychiatric Interview and Wechsler Adult Intelligence Scale‐III, respectively. Associations between the prevalence of diabetes/prediabetes and mental illness/cognitive disability or sociodemographic background were analyzed using the χ^2^‐test.

**Results:**

Seven (6.6%) and 12 (11.3%) participants were diagnosed as having diabetes and prediabetes, respectively, which was a similar trend to that of general populations in Japan National Health and Nutrition Survey data. There was a significant difference in the prediabetes prevalence between groups with and without a history of having social support; however, no significant associations were found between the diabetes/prediabetes prevalence and mental illness/cognitive ability or participants’ sociodemographic background.

**Conclusions:**

The incidence of diabetes in Japanese homeless men was similar to that in the general population, and the prediabetes incidence was lower in the group with social support than in that without. Early intervention for preventing diabetes and social support that focuses on diabetes management is important for homeless people.

## Introduction

Various epidemiological studies have shown a worldwide increase in the prevalence of diabetes mellitus^1^. Patients with diabetes use health services more frequently and for longer; thus, they have more healthcare costs than people without diabetes^2^. Because those with diabetes frequently have major complications, such as coronary heart disease, stroke and nephropathy, they require more healthcare and social support. However, the prevalence of diabetes in homeless people has not been elucidated because of the limited access to this population. Although diabetes patients require integrated multi‐team support, many homeless people stop accessing continuous medical care for two main reasons. The first reason is that most of them have lost their national health insurance, and gaining access to free medical services is a complicated process; they have to visit city hall first to collect a 1‐day free ticket for medical care every time they visit a medical institution. The second reason is that most diabetes patients have no subjective symptoms before the onset of serious diabetic complications. Precise data of the diabetes prevalence might provide evidence for creating a support system for homeless people managing their diabetes.

The only available information about the prevalence of diabetes in the homeless population is two surveys from France^3^ and Ireland^4^, and a meta‐analysis from the USA^5^. To the best of our knowledge, there have been no reports on the diabetes prevalence and related sociodemographic background among the Japanese homeless population.

In the present study, we carried out a survey among homeless men in Nagoya, Japan, to examine the prevalence of diabetes and its association with mental illness, cognitive disability and various social factors.

## Methods

The participants in the present study were recruited in cooperation with the Sasashima Support Center non‐governmental organization on 2 November 2014 in Nagoya, Japan. Although the detailed methodology of the study design has been described previously^6^, the protocol of this survey is described here in brief.

The definition of homelessness used in the present study was according to the United States Department of Health and Human Services’ term “literal homelessness,” which refers to individuals with no stable residence living either in a temporary shelter or unsheltered location not meant for habitation (e.g., the street, a subway station or parked car)^7^. A total of 114 homeless people were screened for this survey. As the number of women participants (7) was too small for a clinically meaningful analysis and discussion, we excluded the data of women. The remaining 106 men with a mean age of 54.2 ± 12.7 years (range 20–78 years) were included in the present analysis.

### Measurements

Participants’ age, residence status, duration and history of homelessness, alcohol consumption, tobacco smoking status, gambling status, history of social support, pension status, and education levels were obtained by medical professionals through interviews. Semi‐structured interviews were carried out by psychiatrists to evaluate participants’ mental status using the Mini‐International Neuropsychiatric Interview according to the *Diagnostic and Statistical Manual of Mental Disorders*, Fourth Edition, Text Revision. Each participant's current mental capacity was assessed by clinical psychologists using the Wechsler Adult Intelligence Scale III simplified version, Dairoku *et al*.'s method^8^, and cognitive disability was defined as an Intelligence Quotient <70. Height, bodyweight, blood pressure and the hematocrit level were also measured, and body mass index was calculated using the following formula: bodyweight (kg) / [height (m)]^2^.

Diabetes screening was carried out by measuring serum hemoglobin A1c (HbA1c) levels according to the criteria of a national survey of public health and nutrition, the Japan National Health and Nutrition Survey^9^, carried out by the Japanese Ministry of Health, Labor and Welfare. Participants who answered, “I have diabetes mellitus” or had an HbA1c value ≥6.5% were considered to have diabetes mellitus, those who had an HbA1c value of 6.0–6.4% were considered to have prediabetes and other participants were considered to be normal. We used the HbA1c level for diabetes screening rather than the result of the 75‐g oral glucose tolerance test in order to minimize participants’ pain burden, and because it was considered very difficult to ensure that participants followed the procedure for the 75‐g oral glucose tolerance test: fasting for >12 h, undergoing blood sampling four times, and abstaining from eating and drinking until the final blood sample was obtained at 120 min after starting the test^10^ because of their socioeconomic status and various degrees of mental health.

Associations between participants’ sociodemographic background or mental illness/cognitive disability, and the prevalence of diabetes and prediabetes were analyzed.

### Statistical analysis

Odds ratios were calculated, and statistical significance was accepted when the 95% confidence interval for the mean difference was not 0, when the 95% confidence interval for the ratio was not 1.0 and when the *P*‐value was <0.05. All statistical analyses were carried out using JMP^®^ software, version 10.0.2 (SAS Institute, Tokyo, Japan).

### Ethical considerations

The present study protocol was approved by the Ethical Review Committee, Graduate School of Medicine, Gifu University on 6 August 2014 (approval no.: 26‐133), and it conforms to the provisions of the Declaration of Helsinki (as revised in Fortaleza, Brazil, October 2013). All participants provided written informed consent. Participants identified as requiring medical treatment were referred on the same day to appropriate medical institutions.

## Results

### Participants’ sociodemographic background

All participants were Japanese in ethnicity and nationality. Most of them lived on the street (63.2%, *n* = 67) for <1 year (54.7%, *n* = 58) without a previous history of homelessness (59.4%, *n* = 63) or an alcohol drinking habit (61.3%, *n* = 65). A total of 28 (26.4%) participants were overweight/obese (body mass index ≥25 kg/m^2^), and just five (4.7%) had a body mass index <18.5 kg/m^2^. A total of 10 (9.4%) participants had a family history of diabetes mellitus (Table 1). Although 53 participants (50.0%) had experience receiving social support, 94 (88.7%) did not receive any pensions. Of 106 participants, 45 (42.4%) were diagnosed as having a mental illness, including schizophrenia, mood disorders, anxiety disorders, personality disorders, and alcohol dependence and/or abuse, 34 (32%) were diagnosed as having a cognitive disability, and 15 (14.2%) participants were diagnosed as having both mental illness and cognitive disability.

**Table 1:** Sociodemographic background of participants

	Total, *n* = 106 (%)
Age (years)	
20–29	5 (4.7)
30–39	11 (10.4)
40–49	22 (20.8)
50–59	27 (25.5)
60–69	31 (29.2)
≥70	10 (9.4)
BMI (kg/m^2^)	
<18.5	5 (4.7)
18.5–24.9	73 (68.9)
≥25.0	28 (26.4)
Family medical history	
Diabetes (+)	10 (9.4)
Hypertension (+)	8 (7.5)
Cancer (+)	16 (15.1)
Mental illness and cognitive disability	
Normal	42 (39.6)
Intellectual disability	19 (17.9)
Mental illness	30 (28.3)
Both	15 (14.2)
Residence	
Street	67 (63.2)
Temporary residence	33 (31.1)
Other	3 (2.8)
Unknown	3 (2.8)
Duration of homeless life (years)	
≤1	58 (54.7)
−2	9 (8.5)
−3	8 (7.5)
−4	5 (4.7)
−5	6 (5.7)
−10	13 (12.3)
≥11	7 (6.6)
History of homelessness (times)	
≤1	63 (59.4)
−2	19 (17.9)
−3	11 (10.4)
−4	3 (2.8)
−5	4 (3.8)
−10	4 (3.8)
≥11	2 (1.9)
Alcohol consumption^a^	
Nothing	65 (61.3)
1	2 (1.9)
2	9 (8.5)
3	2 (1.9)
4–5	3 (2.8)
6–10	8 (7.5)
11–20	8 (7.5)
>20	9 (8.5)
Smoking (no. cigarettes/day)	
Nothing	33 (31.1)
1–10	26 (24.5)
11–20	40 (37.7)
21–30	3 (2.8)
>30	4 (3.8)
History of social support	
(+)	53 (50.0)
(−)	53 (50.0)
Pension	
Nothing	94 (88.7)
Basic pension	4 (3.8)
Employees’ pension	6 (5.7)
Disability pension	0 (0.0)
Others	2 (1.9)
Unknown	0 (0.0)
Education level	
Junior high	48 (45.3)
Senior high	49 (46.2)
College or more	7 (6.6)
History of gambling	
Yes	36 (34.0)
Not now	45 (42.5)
Never	25 (23.6)

a Number of 200‐mL wine glasses/week. BMI, body mass index.

### Prevalence of diabetes and prediabetes among homeless men

The prevalence of diabetes and prediabetes in 106 homeless men were 6.6% (*n* = 7) and 11.3% (*n* = 12), respectively (Table 2). According to the increase of age, the prevalence of prediabetes increased from 0% in those aged 20–29 years to 19.4% in those aged 60–69 years and 20.0% in those aged ≥70 years. Although a diagnosis of diabetes/prediabetes by screening was made based on the HbA1c levels, not results of the 75‐g oral glucose tolerance test, no participants showed a discrepancy between blood glucose and HbA1c levels (Table 2). In addition, no participants had severe anemia that might affect HbA1c levels. The mean ± standard deviation of participants’ hematocrit level was 41.9 ± 3.4% (minimum 34.6%, maximum 48.8%).

**Table 2:** Prevalence of hemoglobin A1c and blood glucose levels in each generation

Age (years)	Total			20–29			30–39			40–49			50–59			60–69			≥70		
HbA1c(%)	Prevalence		Blood glucose	Prevalence		Blood glucose	Prevalence		Blood glucose	Prevalence		Blood glucose	Prevalence		Blood glucose	Prevalence		Blood glucose	Prevalence		Blood glucose
	*n*	%	Mean ± SD min–max	*n*	%	Mean ± SD min–max	*n*	%	Mean ± SD min–max	*n*	%	Mean ± SD min–max	*n*	%	Mean ± SD min–max	*n*	%	Mean ± SD min–max	*n*	%	Mean ± SD min–max
≤5.9	87	82.1	94.60 ± 16.13	5	100.0	80.80 ± 5.93	9	81.8	88.56 ± 12.73	19	86.4	87.05 ± 13.20	23	85.2	96.04 ± 19.42	23	74.2	102.04 ± 12.02	8	80.0	102.38 ± 17.59
			65–160			75–89			74–111			65–112			77–160			78–126			82–127
6.0–6.4	12	11.3	127.75 ± 64.47	0	0.0	–	1	9.1	100	1	4.5	86	2	7.4	105.50 ± 0.71	6	19.4	154.17 ± 85.09	2	20.0	105.50 ± 28.99
			85–324			–			100–100			86–86			105–106			99–324			85–126
≥6.5	7	6.6	124.29 ± 50.34	0	0.0	–	1	9.1	89	2	9.1	153.50 ± 72.83	2	7.4	101.50 ± 12.02	2	6.5	135.50 ± 74.25	0	0.0	–
			83–205			–			89–89			102–205			93–110			83–188			–
Total	106	100.0	100.31 ± 30.74	5	100.0	80.80 ± 5.93	11	100.0	89.64 ± 11.89	22	100.0	93.05 ± 28.01	27	100.0	97.15 ± 18.24	31	100.0	114.29 ± 44.26	10	100.0	103.00 ± 18.32
			65–324			75–89			74–111			65–205			77–160			78–324			82–127

HbA1c, hemoglobin A1c; min–max, minimum to maximum; SD, standard deviation.

### Diabetes or prediabetes prevalence and sociodemographic background

The distributions of 106 participants in each diabetic category (diabetes mellitus, prediabetes and normal) stratified according to various mental/cognitive and sociodemographic factors are shown in Table 3. The prevalence of prediabetes was significantly higher in the group without social support than in that without social support. There were no significant associations between the prevalence of diabetes/prediabetes and other factors, such as having a mental illness/cognitive disability, residence, current duration and previous experience with homelessness, lifestyle habits (e.g., smoking and alcohol consumption), and education levels.

**Table 3:** Participants’ diabetes prevalence according to hemoglobin A1c levels, stratified by background

HbA1c (%)	≤5.9		6.0–6.4		≥6.5		Total	
	*n*	%	*n*	%	*n*	%	*n*	%
Total	87	82.1	12	11.3	7	6.6	106	100.0
Mental illness/cognitive disability								
Normal	34	81.0	5	11.9	3	7.1	42	100.0
Cognitive disability	16	84.2	2	10.5	1	5.3	19	100.0
Mental illness	26	86.7	3	10.0	1	3.3	30	100.0
Cognitive disability + mental illness	11	73.3	2	13.3	2	13.3	15	100.0
Residence								
Street	54	80.6	8	11.9	5	7.5	67	100.0
Others	30	83.3	4	11.1	2	5.6	36	100.0
Duration of homelessness (years)								
1	50	86.2	5	8.6	3	5.2	58	100.0
≥2	37	77.1	7	14.6	4	8.3	48	100.0
Past experience of homelessness (times)								
1	52	82.5	6	9.5	5	7.9	63	100.0
≥2	35	81.4	6	14.0	2	4.7	43	100.0
Alcohol consumption								
−	51	78.5	8	12.3	6	9.2	65	100.0
+	36	87.8	4	9.8	1	2.4	41	100.0
Tobacco smoker								
−	27	81.8	3	9.1	3	9.1	33	100.0
+	60	82.2	9	12.3	4	5.5	73	100.0
Gambling								
−	24	96.0	1	4.0	0	0.0	25	100.0
+	27	75.0	6	16.7	3	8.3	36	100.0
+ Previously, not now	36	80.0	5	11.1	4	8.9	45	100.0
Experience with social support								
−	40	75.5	10	*18.9	3	5.7	53	100.0
+	47	88.7	2	*3.8	4	7.5	53	100.0
Pension								
−	77	81.9	10	10.6	7	7.4	94	100.0
+	10	83.3	2	16.7	0	0.0	12	100.0
Education level								
Less than senior high school	47	82.5	5	8.8	5	8.8	57	100.0
Senior high school or more	38	80.9	7	14.9	2	4.3	47	100.0

χ^2^‐tests *P <* *0.05. HbA1c, hemoglobin A1c.

## Discussion

Diabetes is a global health problem causing increased morbidity and mortality; therefore, the prevention, early detection and appropriate treatment are required for all populations. The number of people with diabetes is projected to increase in developed and developing countries^1^. This trend has been more serious in Asia than in Europe or North America^11^, as East Asian people, including the Japanese, have lower levels of insulin secretion than Caucasians^12^.

Homelessness is a worldwide social and health issue, with mortality among homeless people being substantially higher, even in developed countries with high‐quality social support systems^13^; the situation might be worse in countries with fewer organized welfare systems. Low‐income people with poor mental/physical health are likely to experience homelessness in their lives^14^, and homelessness is an independent risk factor for deaths from specific causes^15^. However, there is little information about diabetes in homeless people.

In the present study, we showed that 6.6% and 11.3% of our sample of homeless Japanese men living in an urban city area had diabetes and prediabetes, respectively. These numbers were almost the same as those in the general population of a national survey among people aged 20–59 and ≥70 years; however, the prevalence was relatively lower in homeless Japanese men than in the general population aged 60–69 years^9^. A survey in Paris showed that the estimated prevalence of diabetes in homeless people was 6.2%^3^. A study of Irish homeless people showed that 10% and 21% of the study population had diabetes and prediabetes, respectively^4^. The present study is the first to show the diabetes/prediabetes prevalence in an Asian homeless population. The data presented herein will be a valuable resource for health policymakers in providing health support for homeless people in Japan and Asia. Tsai *et al*.^14^ also advocated that obtaining accurate prevalence estimates is important to progress social support, including allocation of governmental resources. Although there are barriers to accessing medical care services in the homeless population, preventive programs for diabetes might be cost‐effective if targeted at the homeless population with a high diabetes/prediabetes prevalence that is almost the same level as that in the national survey data in Japan. The social welfare service served high‐energy meals that were mainly popular items, such as curry and rice or meat and vegetables over rice, at least twice a day. Therefore, most participants might have had almost the same nutrition level as the general population, and this might be why the prevalence of diabetes in homeless people was the same as that in national survey data. No participants were malnourished, and 73 (68.9%) and 28 (26.4%) participants were normal and overweight/obese, respectively. Diabetes is a very costly disease once diabetic complications develop, including blindness induced by diabetic retinopathy, amputation induced by diabetic neuropathy and hemodialysis induced by diabetic nephropathy, which require huge budgets for social support and medical expenditure^2^. If diabetes patients without medical care reach the requirement for hemodialysis therapy, they will incur substantial medical costs until the end of their life. Conversely, diabetes patients taking only oral medication will incur this medical expense in the long term, but their life‐long medical expenditure might be far less than that for hemodialysis per year. The preventive approach for diabetes should be as cost‐effective for homeless people as it is for the general population. On the basis of the same viewpoint, Morrison indicated that homelessness is more hazardous than being in conventional deprived socioeconomic circumstances, and homeless people might benefit from more intensive targeted health and social interventions^15^.

We showed a significant relationship between previous experiences of social support and diabetes/prediabetes prevalence, indicating that interventions improving physical health with social support might be effective for directly or indirectly preventing diabetes. We previously reported that the high percentage of mental illness and cognitive disability in Japanese homeless people^6^ was similar to that of homeless people in Western countries^16^, ^17^. Coordinated programs for diabetes prevention/treatment with mental health and cognitive support should be required for homeless people. Effective interventions could improve health and increase access to medical institutions among homeless populations, as Fitzpatrick‐Lewis *et al*. showed^18^. Previous review papers emphasized that interventions to improve health through the coordinated support for access to healthcare was found to be important^16^, ^17^, ^18^ because of the high prevalence of mental health problems in this population. Interventions to access social support and medical care might improve the health condition of homeless people, thereby facilitating their transition back to a stable social life and potentially decreasing their prevalence of diabetes.

The present study had a limitation. We carried out a cross‐sectional, single‐day survey design and participants were recruited from a single site, although the majority of homeless people live near Nagoya station area. Therefore, we could not confirm whether social support intervention could prevent diabetes mellitus. To overcome this limitation, an additional follow‐up study would be required.

The prevalence of diabetes among homeless men in Japan was almost similar to that of the general population; thus, early detection and effective intervention for diabetes/prediabetes might be required in the homeless population.

## Disclosure

The authors declare no conflict of interest.
